# Supplementary material for: Fatty Acid and Amino Acid Profiles of Seven Edible Insects: Focus on Lipid Class Composition and Protein Conversion Factors
Source: Foods. 2023 Nov 10;12(22):4090. doi: 10.3390/foods12224090 (PMC10670213; doi:10.3390/foods12224090)
Supplement: Supplementary file 1 [file foods-12-04090-s001.zip › foods-2689110-supplementary.pdf]

Supplementary Table S1. Proximate composition of edible insects, expressed as g / 100 g DM (mean ± SEM), except for moisture, which is expressed as g / 100 g insect.

	**ACD**	**ALD**	**BD**	**GM**	**LM**	**TM**	**ZM**
**Total lipids**	21.32±0.36^{b}	21.82±0.30^{b}	13.96±0.45^{a}	53.63±0.80^{d}	32.28±0.43^{c}	22.61±0.64^{b}	33.97±0.82^{c}
Neutral lipids	9.94±0.33^{ab}	10.32±0.39^{ab}	7.91±0.53^{a}	46.22±0.88^{d}	24.44±0.55^{c}	10.79±0.68^{b}	22.18±0.98^{c}
Glycolipids	5.85±0.18^{b}	8.32±0.54^{c}	2.64±0.22^{a}	5.97±0.32^{b}	3.08±0.31^{a}	9.58±0.54^{cd}	10.61±0.78^{d}
Phospholipids	5.54±0.29^{c}	3.18±0.41^{b}	3.40±0.39^{b}	1.43±0.12^{a}	4.76±0.22^{c}	2.23±0.28^{ab}	1.17±0.28^{a}
**True protein**	39.37±0.10^{c}	55.74±0.21^{e}	61.49±0.27^{f}	28.69±0.53^{a}	44.23±0.59^{d}	44.68±0.74^{d}	34.57±0.20^{b}
**Moisture**	68.51±0.13^{d}	72.5 ±0.06^{e}	64.10±0.72^{c}	56.21±1.77^{a}	62.45±0.15^{bc}	63.00±0.17^{bc}	61.37±0.06^{b}

$^{a–d}$ Mean values in the same row not followed by a common letter differ significantly ($p < 0.001$). ACD: *A. domesticus;* ALD: *A. diaperinus;* BD: *B. dubia*; GM: *G. mellonella;* LM: *L. migratoria;* TM: *T. molitor;* ZM: *Z. morio.*
